# Reannotation and extended community resources for the genome of the non-seed plant *Physcomitrella patens* provide insights into the evolution of plant gene structures and functions

**DOI:** 10.1186/1471-2164-14-498

**Published:** 2013-07-23

**Authors:** Andreas D Zimmer, Daniel Lang, Karol Buchta, Stephane Rombauts, Tomoaki Nishiyama, Mitsuyasu Hasebe, Yves Van de Peer, Stefan A Rensing, Ralf Reski

**Affiliations:** 1 Plant Biotechnology, Faculty of Biology, University of Freiburg, Schaenzlestrasse 1, 79104, Freiburg, Germany; 2 Institute of Biology II, Faculty of Biology, University of Freiburg, Schaenzlestrasse 1, 79104, Freiburg, Germany; 3 BIOSS - Centre for Biological Signalling Studies, Freiburg, Germany; 4 FRIAS - Freiburg Institute for Advanced Studies, Freiburg, Germany; 5 Advanced Science Research Center, Kanazawa University, Kanazawa, Japan; 6 National Institute for Basic Biology, Okazaki, Japan; 7 Faculty of Biology, University of Marburg, Karl-von-Frisch-Str. 8, 35043, Marburg, Germany; 8 Department of Plant Systems Biology, VIB, Technologiepark 927, B-9052, Ghent, Belgium; 9 Department of Plant Biotechnology and Bioinformatics, Ghent University, Technologiepark 927, B-9052, Ghent, Belgium; 10 School of life Science, The Graduate University for Advanced Studies, Okazaki 444-8585, Japan

**Keywords:** Bryophyte, Physcomitrella patens, Genome annotation, Gene structure, Reference genome, Model organism, UTR, Plant evolution, Non-flowering plant, Orphan genes

## Abstract

**Background:**

The moss *Physcomitrella patens* as a model species provides an important reference for early-diverging lineages of plants and the release of the genome in 2008 opened the doors to genome-wide studies. The usability of a reference genome greatly depends on the quality of the annotation and the availability of centralized community resources. Therefore, in the light of accumulating evidence for missing genes, fragmentary gene structures, false annotations and a low rate of functional annotations on the original release, we decided to improve the moss genome annotation.

**Results:**

Here, we report the complete moss genome re-annotation (designated V1.6) incorporating the increased transcript availability from a multitude of developmental stages and tissue types. We demonstrate the utility of the improved *P. patens* genome annotation for comparative genomics and new extensions to the cosmoss.org resource as a central repository for this plant “flagship” genome. The structural annotation of 32,275 protein-coding genes results in 8387 additional loci including 1456 loci with known protein domains or homologs in Plantae. This is the first release to include information on transcript isoforms, suggesting alternative splicing events for at least 10.8% of the loci. Furthermore, this release now also provides information on non-protein-coding loci. Functional annotations were improved regarding quality and coverage, resulting in 58% annotated loci (previously: 41%) that comprise also 7200 additional loci with GO annotations. Access and manual curation of the functional and structural genome annotation is provided via the http://www.cosmoss.org model organism database.

**Conclusions:**

Comparative analysis of gene structure evolution along the green plant lineage provides novel insights, such as a comparatively high number of loci with 5’-UTR introns in the moss. Comparative analysis of functional annotations reveals expansions of moss house-keeping and metabolic genes and further possibly adaptive, lineage-specific expansions and gains including at least 13% orphan genes.

## Background

Given its phylogenetic key position as an early diverging land plant that bridges the gap of about one billion years between the unicellular green algae and flowering plants, the moss *Physcomitrella patens* (Physcomitrella) unites most of the attributes desirable for a model organism, including a short generation time, small stature, comparatively low morphological complexity, a haplo-dominant life cycle, traceable cell lineage, high growth rate and simplicity of genetic transformation. Combined with the potential for evolutionary-developmental (evo-devo) studies, these traits have become increasingly attractive to a wide range of plant scientists. Over the last two decades, a growing community has established *P. patens* as a model organism with a well-developed molecular toolbox including the uniquely efficient gene targeting via homologous recombination and comprehensive genomics resources which have been made available early on using the central web service cosmoss.org [1-4]. The moss is also a promising model for green biotechnology [5-9], which allows the production of safe recombinant proteins with eukaryotic post-translational modifications in competitive quantities.

The draft genome sequence of the moss *Physcomitrella patens* was published in 2008 [10]. The availability of the genomic sequence and the established molecular toolbox provide the ideal foundation for extensive comparative and evo-devo analyses studies. This is reflected in the publication record - a growing body of researchers from all fields has begun to apply Physcomitrella as an additional model organism for comparative studies [11-20]. Various evo-devo studies have demonstrated the ability of Physcomitrella transgenes to act as functional orthologs in cross-species complementation assays using *A. thaliana* mutant lines (e.g. [21-24]). Additionally, large-scale analyses and cross-kingdom comparisons increasingly utilize the moss as a representative organism for the plant kingdom [25-29]. This ongoing interest, the available resources, the active community, and the moss’ attractive phylogenetic position recently led the U.S. Department of Energy’s Joint Genome Institute (JGI) to select *P. patens* as a “plant flagship genome” [30].

The quality of a genome annotation is the bottleneck for any form of downstream and comparative analyses. Particularly affected by flaws are the large-scale, high-throughput approaches employed in systems biology [31]. Following its initial V1.0 annotation, the *P. patens* genome annotation has been iteratively improved. The draft V1 assembly was based on whole-genome shotgun Sanger sequencing at 8.6x clone depth and comprises 2536 V1 scaffolds. This number was reduced to 2106 in the released V1.1 after removal of bacterial contaminations [10]. After an additional round of scaffold filtering, released as V1.2, the genome sequence of the 27 chromosomes is still scattered over 1995 genomic scaffolds [3]. The filtering of the gene catalogue, in particular by removal of transposable elements and other non-protein coding regions, led to the prediction of 27,966 protein-coding genes [3]. The results of a survey conducted on behalf of the JGI at the annual moss meeting 2011 clearly show that for most groups (79%) the initial sequencing and release of the genome was already “essential”. However, many research topics were listed that “would be enabled if a highly complete and accurate reference genome for Physcomitrella was available”, revealing the need for advanced genome annotation. For example, there were cases of well-characterized moss genes that were present in the genomic sequence but were missing from the gene catalogue [32]. In V1.2 only 4515 (~16%) gene models had both 5’ and 3’-UTRs (untranslated regions). Over 23,000 genes missed either 5’-UTR or 3’-UTR annotation and thus were incomplete. Furthermore, functional annotation was only available for 41% of the genes and hardly any of these annotations were backed by traceable experimental evidence. A further shortcoming was that no established and universal means for scientists and curators existed to link (published) knowledge on moss genes to the digital representations scattered across several databases.

Model organism databases (MODs, e.g. Gramene [33], TAIR [34], FlyBase [35]) are integrated, web-accessible resources, which are prerequisite for the success and the quality of a reference genome [36]. They act as general repositories for all kinds of research data and scientific knowledge that is generated by the scientific community. Thus, they provide the necessary infrastructure for researchers working with model species, are the focal point for scientists new or outside the field and provide conceptual interfaces for data exchange with more general data repositories (e.g. NCBI, UniProt [37], Ensembl [38], Phytozome [39], and PLAZA [40]) to enable comparative analyses and ensure overall data quality [41,42].

Experience from various MODs and whole genome sequencing projects shows that automatic annotation without substantial manual curation is not sufficient to ensure data quality and knowledge discovery [43]. An active community is necessary to transfer all available data, especially the biological knowledge covered by the scientific literature, to the genome annotation [44].

Initially, the cosmoss.org resource was set up to provide access to the *P. patens* virtual transcriptome assemblies and annotation [1] using BLAST services, keyword search and sequence retrieval. Subsequently, it was extended to provide services for splice site prediction [2], for mining gene families of transcription associated proteins (PlanTAPDB; [45]) and to predict dual protein targeting (ATP; [46]). Since the release of the initial *P. patens* genome assembly, the resource cosmoss.org additionally provides access to the draft genome sequence [10], the genetic map [47], and the filtered genome annotation V1.2 [3]. Moreover, cosmoss.org serves as a platform to coordinate the analysis and annotation of the *P. patens* genome sequence. As part of this, a wiki and several mailing lists have been set up to report and discuss the results within the community. Additionally, an integrative genome browser serves as a main entrance point for the exploration of the moss genome and annotation. The integrative cosmoss.org browser is based on the Gbrowse software [48] and provides base pair level resolution for large-scale annotation data covering predictions for all different kinds of genomic regions ranging from protein-coding genes, transposable elements and repeats to tRNA, rRNA, miRNAs, and other non-protein coding RNAs. Furthermore, the annotations are linked to the cosmoss.org internal annotation resources as well as to GenBank [49], Pfam [50], miRBase [51] and comparative genomics resources like Phytozome [39] and PLAZA [40].

While a significant part of cosmoss.org is based on our analyses, we are continuously integrating external published data, e.g. sRNAs [52], miRNAs [53] and EST (expressed sequence tag) or short read data from the sequence read archive (SRA [54]) and from collaborators around the world. In addition, the cosmoss.org gene annotation releases are shared and hosted at the NCBI and the comparative plant resources, Phytozome, PLAZA, and PlantGDB [55]. In July 2009, the Physcomitrella community annotation services were transferred from the JGI website to cosmoss.org and the resource now functions as the central annotation repository for the moss *P. patens*.

Here, we report the complete re-annotation of the *P. patens* genome assembly V1 (Table 1), demonstrate its utility for comparative analyses and introduce the extensions to the cosmoss.org resource to act as a permanent central genome annotation repository and model organism database involving:

• The improvement of gene structures with specific focus on the incorporation of transcript evidence to cover alternative splice variants and to derive UTRs.

• Complete renewal of the functional annotation.

• Prediction and annotation of non-protein-coding genes.

• Integration of user annotations: Structural and functional annotations.

• Integration of manual annotations: Development of community annotation services.

**Table 1:** ***P. patens*genome annotation releases**

		**Rensing et al.**	**Lang et al.**	**V1.6**
		**(01/2008) V1.1**	**(10/2008) V1.2**	**cosmoss.org**
**genome size (Mb)**		480	480	480
**scaffolds**		2,106	1,995	1,985
**protein-coding genes**		35,938	27,966 (-7,972)	32,275 (+4,309)
**protein-coding genes with EST support**		12,593	19,119 (+6,523)	26,722 (+7,603)
**protein-coding transcripts**		35,938	27,966 (-7,972)	38,357* (+10,391)
**annotated as alternatively spliced**		-	-	3,500
**genes with UTRs**		4,517	4,515 (-2)	15,757* (+11,242)
**either UTR**	**genes**	8,418	8,381 (-37)	16,010 (+7,629)21,464*
	**transcripts**			
**gene density (kb per gene)**		13.4	17.2	14.9*
**exons / gene**		4.9	5.4	5.0*
**mean exon length (bp)**		246	234	275*
**mean intron length (bp)**		311	277	278*
**Gene structures altered since previous release:**				
**Models updated**			-	22,307
**Models identical**			27,966	1,582
**Models added**			-	8,387
**Loci added with plant homologs**			-	1,338
**Models added with Pfam domain**				2,196
**Loci added with Pfam domain**				1,456
**Models filtered out**			7,972	4,077
**miRNA families**			99	108&
**miRNAs**			220	229&
**tRNA genes**			432	432
**rDNA regions**			798	798
**snRNAs**			213	213
**Eukaryotic type signal recognition particle RNA (SRP)**			6	6

*including splice variants &data from the miRBase registry, release 18 [59].

## Results and discussion

### Improved structural annotation of the *P. patens* genome

The cosmoss.org *Physcomitrella patens* V1.6 genome annotation reported here is the result of iterative rounds of evidence mapping, repeat masking, gene structure prediction, filtering and model selection and harbors annotation of protein-coding genes, transposable and repetitive elements and, for the first time, definition of non-protein-coding loci. The release comprises 32,275 protein-coding genes, 432 tRNA loci, 798 rDNA regions, 229 miRNA precursors (108 families) [51], 213 snRNA genes, and 6 SRP (signal recognition particle) loci. Considering the number of miRNA families, *P. patens* with 108 families has an intermediate position between the green alga *C. reinhardtii* (47 families) and the flowering plant *A. thaliana* (187 families) [51]. Consistent with previous findings [3,10] about half of the genome consists of full length LTR retrotransposons and related fragments including chromodomain-containing gypsy LTR retrotransposons (Tcn1) shared by fungi and non-flowering plants [56].

V1.6 protein-coding gene predictions are based on multiple sources of evidence. Of prime importance are ESTs (Additional file 1: Table A1) from 19 different experimental conditions, tissue types and developmental stages providing a reliable basis for gene structure prediction. The combined transcript evidence was used to train species-specific prediction models using SpliceMachine [57] and EuGène [58], followed by the generation of weighted consensus gene structures using EVidenceModeler [59] and PASA [60]. The prediction procedure was repeated iteratively. Each round involved several filtering steps for separation of non-protein coding, repeat and transposable element-associated genes. The interim versions V1.3-1.5 were not published but are the basis for V1.6 (see Additional file 2: Table A2). The whole protein-coding gene prediction and annotation process is summarized in Additional file 3: Figure A1. The annotation release V1.6 comprises 32,275 loci coding for 38,357 protein-coding transcripts (see annotation releases overview Table 1). 26,722 (~83%) loci are supported by transcript evidence (i.e. EST or full-length cDNA). The average V1.6 gene has a mean length of 2369 bp and a transcript length of 1389 bp. The number of only 1582 unchanged gene models from V1.1 to V1.6 is an excellent indicator of the extent of changes and improvements that led to the current release. While the changes in release V1.2 (Table 1; [3]) were restricted to the removal/filtering of non-protein-coding genes (7972 filtered models), the complete annotation process leading to V1.6 (Additional file 3: Figure A1) resulted in 22,307 (~80% of V1.2) updated models, 8387 (25% of V1.6) new loci and 4077 (15% of V1.2) models which have been removed due to non-protein coding/transposable element origin. Of the new loci, 1535 transcripts (1338 genes) are part of a gene family with at least one additional plant species, and 2196 transcripts (1456 genes) encode at least one Pfam domain. Also, published genes and gene models released by the scientific community were mapped, manually curated and integrated into V1.6.

Inspection of UTR annotations indicates that gene structure completeness is much improved in V1.6. The number of protein-coding loci with both 5’ and 3’ UTRs were increased from 4515 to 15,757 transcripts. The median transcript length increased from 987 (V1.2) to 1248 bases (V1.6) which can be explained by a higher percentage of annotated UTR regions in this release. V1.6 gene models only contain UTR annotations if they are fully supported by transcript evidence.

**Figure 1:**
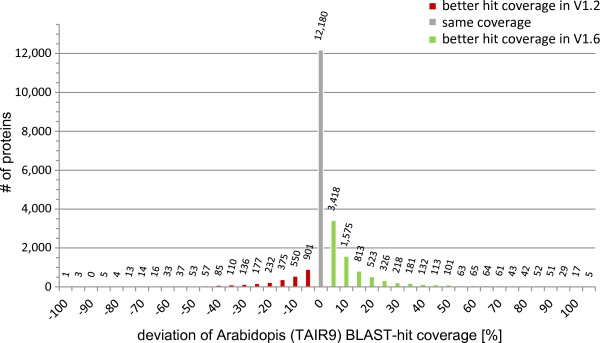
***A. thaliana *best hit (BLASTP) coverage changes from *P. patens *V1.2 to V1.6 34.5 % of the protein-coding gene models (V1.6) covers better their closest *A. thaliana *homolog.**

To further assess model quality and completeness we compared predicted protein sequences from V1.2 and V1.6 based on the coverage of their respective closest homolog in *A. thaliana* (Figure 1). The direct comparison demonstrates V1.6 models generally as more complete than those in V1.2: 34.5% of the V1.6 proteins with a corresponding V1.2 model cover a higher proportion of the length of their best *A. thaliana* BLASTP hit.

### Annotation and characterization of alternative splicing

We used the PASA pipeline [60] to study the extent and characteristics of alternative splicing (AS) in the moss. We used GenomeThreader [61] transcript alignments in exchange of PASA’s standard GMAP alignments [62] as GenomeThreader supports alignment of transcript evidence to multiple (nearly identical) loci. This is important for covering nearly identical tandemly-arrayed genes observed in *P. patens*[3,10] and of other segmental duplications.

In total, the PASA pipeline reported 39,106 genes (subclusters) out of which 6556 (16.8%) show AS (Table 2). Consistent with previous analyses [10], we find alternative acceptors as the most prevalent event of AS (4443 events) in PASA assemblies. Among gene loci with alternate transcripts, intron retention is the most frequent form (~40% of all genes). Analysis based on large-scale EST libraries largely derived from total RNA preparations, undoubtedly include partially spliced intermediates which might lead to an overestimation of the fraction of intron retention variants, however our data reflect the findings observed for other plants where intron retention is the preferred form of AS, followed by an alternative acceptor [63-65]. Based on the Sanger EST data the percentage of genes with evidence for AS in *P. patens* (21%) is in the range of flowering plants like *A. thaliana* (20%) and *Oryza sativa* (30%). Given the fact that recently the use of next generation deep sequencing data resulted in a substantial upward revision of the number of alternatively spliced genes in *A. thaliana* to at least 42% [66] we expect the true extent of AS to be similar in the moss. This is also supported by the extent of NAGNAG alternative splicing based on NGS data which is in the same range as in flowering plants [65].

**Table 2:** **Summary statistics of alternative splicing in *P. patens *genes**

	**# Assemblies**	**% Assemblies**	**# Sub clusters**	**% Sub clusters**	**# Genes**	**% Genes**
**involved in alt-splicing**	12,941	27.21	5,195	13.28	5,177	14.65
**alt_acceptor**	4,443	34.33	1,649	31.74	1,657	32.01
**alt_donor**	3,940	30.45	1,414	27.22	1,433	27.68
**alternate_exon**	2,423	18.72	886	17.05	908	17.54
**ends_in_intron**	989	7.64	836	16.09	836	16.15
**retained_exon**	800	6.18	552	10.63	574	11.09
**retained_intron**	2,692	20.8	2,055	39.56	2,082	40.22
**skipped_exon**	810	6.26	552	10.63	575	11.11
**spliced_intron**	3,523	27.22	2,055	39.56	2,080	40.18
**starts_in_intron**	1,162	8.98	1,010	19.44	1,033	19.95

Overview of the alternative splicing events observed in *P. patens* using the PASA software and ~300,000 ESTs.

In V1.6 we have incorporated information on AS for the first time into the released genome annotation. The integration of AS into the annotation process leads to 3500 (10.8%) annotated loci with an average of 2.52 transcripts per locus and a maximum of 11 transcripts in V1.6. 1775 loci (51% of AS transcripts; 5.5% of all genes) have an altered coding sequence (CDS) due to alternative splicing resulting in 2380 distinct proteins. In contrast to *A. thaliana*, where the analysis of large-scale full-length cDNAs suggested most splicing events to occur outside of coding regions in the 5’-UTR [67], alternative splicing in moss seems to affect UTRs and CDS regions to a similar degree. 2948 alternative transcripts are due to alternative splicing in the UTRs of 1991 loci (56% of AS transcripts; 6.2% of all genes). Extensive alternative splicing of moss 5’-UTRs was observed previously in an individual study of the MDHAR genes [68].

### Insights into the evolution of gene structures along the green lineage

The advanced annotation enabled us to address comparative questions on genome and gene structure evolution in plants. A comprehensive overview of genome and protein-coding gene statistics of several Viridiplantae species is compiled in Table 3 and Additional file 4: Table A3. Our complete re-evaluation of the respective genome annotations supports previous observations, but also provides novel insights. The average volvocine green algal gene is about twice the length of the average land plant gene [69]. This trend is not as pronounced in the transcript and CDS lengths, but still the average algal transcript is longer than those of the land plants (Table 3). While the median exon length is similar in all seven Viridiplantae species, Chlamydomonas introns are longer and more numerous than those of land plants ([69]; Table 3).

**Table 3:** Protein-coding gene statistics of selected Viridiplantae

		***C. reinhardtii***	***P. patens***	***P. patens***	***S. moellendorffii***	***O. sativa***	***A. thaliana***
		**(V4.1)**	**(V1.2)**	**(V1.6)**	**(FM3)**	**(V6.1)**	**(TAIR10)**
Genes	#	15,935	27,726	32,275	22,259	40,577	27,206
Transcripts	#	15,935	27,966	38,357	22,259	50,939	35,176
Gene length [bp]	x̄	5,363	2,499	2,369	1,699	2,816	2,190
	x̃	4,273	1,878	1,809	1,368	2,148	1,896
Transcript length [bp]	x̄	2,898	1,269	1,389	1,194	1,540	1,540
	x̃	2,284	987	1,248	987	1,395	1,388
CDS length [bp]	x̄	2,043	1,131	1,062	1,145	1,079	1,234
	x̃	1,425	867	813	951	879	1,053
Exon length [bp]	x̄	322	234	275	213	313	261
	x̃	155	145	155	128	158	147
Intron length [bp]	x̄	308	277	278	110	415	164
	x̃	238	206	213	59	169	100
Exons per gene	x̄	9.0	5.4	5.0	5.6	4.9	5.9
	x̃	7	4	3	4	3	4
Introns per gene	x̄	8.0	4.4	4.0	4.6	3.9	4.9
	x̃	6	3	2	3	2	3
5'-UTR exon length [bp]	x̄	181	171	211	92	189	119
	x̃	138	127	157	50	122	88
5'-UTR intron length [bp]	x̄	513	502	520	184	666	315
	x̃	274	353	390	70	355	239
3'-UTR exon length [bp]	x̄	634	323	338	166	377	217
	x̃	537	299	311	110	316	201
3'-UTR intron length [bp]	x̄	859	505	268	280	500	204
	x̃	470.5	215	213	65	180	104
5'-UTR length [bp]	x̄	204	231	307	110	254	152
	x̃	158	184	258	54	156	112
3'-UTR length [bp]	x̄	653	352	367	194	464	237
	x̃	551	322	334	121	358	210
Multi exon transcript	#	15,322	23,758	29,378	18,789	40,859	29,050
	%	96.1%	84.9%	76.6%	84.4%	80.2%	82.6%
Single exon transcript	#	613	4,208	8,979	3,470	10,080	6,126
	%	3.9%	15.1%	23.4%	15.6%	19.8%	17.4%
Transcripts with both 5' and 3'-UTR	#	15,856	4,515	15,757	2,178	31,089	26,255
Transcripts with 5'-UTR	#	15,896	5,691	18,180	2,506	31,793	27,097
Transcripts with 3'-UTR	#	15,895	7,205	19,041	3,653	33,252	28,049
Transcripts without UTR	#	0	19,585	16,893	18,278	16,983	6,285
Multi exon 5'-UTR	#	1,743	1,556	7,120	399	7,940	6,486
	%	11.0%	27.3%	39.2%	15.9%	25.0%	23.9%
Single exon 5'-UTR	#	14,153	4,135	11,060	2,107	23,853	20,611
	%	89.0%	72.7%	60.8%	84.1%	75.0%	76.1%
Multi exon 3'-UTR	#	480	462	1,387	522	5,010	2,027
	%	3.0%	6.4%	7.3%	14.3%	15.1%	33.1%
Single exon 3'-UTR	#	15,415	6,743	17,654	3,131	28,242	26,022
	%	97.0%	93.6%	92.7%	85.7%	84.9%	92.8%

Selected properties of structure and organization of protein-coding genes within Viridiplantae. A more detailed list can be found in Additional file 4: Table A3. x̄ - average x̃ - median # - amount.

**Figure 2:**
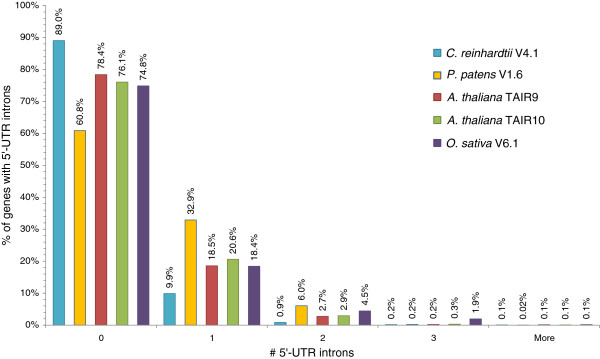
**Comparison of 5’-UTR intron numbers in Viridiplantae5’-UTR intron number frequencies of selected Viridiplantae genomes.** The y-axis labels give the number of transcripts w/o 5’UTR introns in percentage of all transcripts with 5’UTR.

Corresponding with its intermediary phylogenetic position, the median intron length of the average *P. patens* gene is intermediate (Table 3) between that of algae and flowering plants, implicating a trend towards shorter introns during the evolution of land plants. Whereas the median algal 5’-UTR length are in the range of Tracheophyta, the median moss 5’-UTR length is about 100 bp longer. Moss 5’-UTR regions more frequently comprise multiple exons than other plant genomes, with 40% of genes so annotated. Additionally, although there are substantially more predicted transcripts in *O. sativa* and more transcripts with 5’-UTR introns (8227), the total number of transcripts with multiple 5’-UTR exons in *P. patens* is nearly as high (7120) (p-value < 2E-238); Figure 2). This will likely increase as further high-resolution transcriptome data are obtained, since only 18,180 moss transcripts currently have annotated 5’UTRs compared with 31,793 transcripts in the rice annotation.

**Figure 3:**
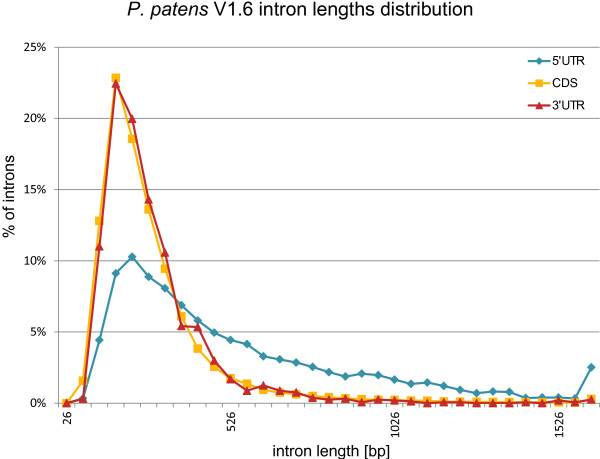
***P. patens *V1.6 intron lengths distribution 5’-UTR, CDS, and 3’UTR intron lengths in comparison.** The percentage of introns longer than 500 bp is much higher in 5’-UTRs than in CDS and 3’-UTR introns.

In *A. thaliana* introns in the 5’-UTRs tend to be longer than introns in the coding sequence and 3’-UTRs [70]. A plot of *P. patens* intron lengths (Figure 3) reveals a similar distribution. *P. patens* 5’-UTR introns are significantly longer than introns in the CDS (p-value = 0) and 3’-UTR (p-value <1.03E-193). In particular, the percentage of introns longer than 500 bp is much higher in the 5’-UTRs than in the CDS or in the 3’-UTRs. The mean 5’-UTR intron length for *P. patens* is 520 bp, whereas it is 264 bp for the CDS and 268 bp for 3’-UTR introns.

**Figure 4:**
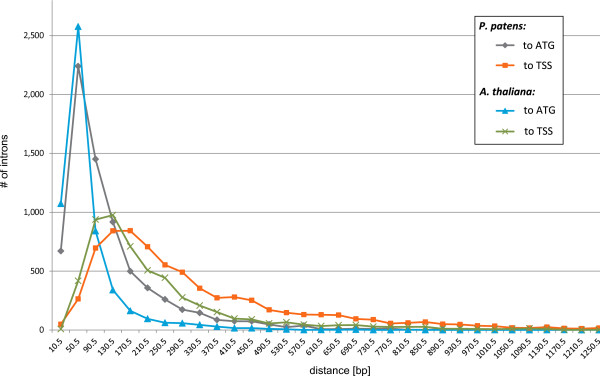
**Distance to translation and transcription start sites of 5’-UTR intron positions Distribution of 5’-UTR positions for *P. patens *and *A. thaliana *transcripts in comparison.** The closeness of 5’-UTR to the initiating ATG is more pronounced in *A. thaliana*. While ~75% of introns are closer than 65 bp in *A. thaliana* only ~50% are in *P. patens*.

Furthermore, in *A. thaliana* the 5’-UTR introns are preferentially located close to the initiating ATG codon [70]. A comparative distribution plot of the proximate 5’-UTR intron distance to the start of transcription and translation reveals similar distributions in *P. patens* and *A. thaliana* (Figure 4). Certainly the closeness of 5’-UTR introns to the initiating ATG is more pronounced in *A. thaliana* (*A. thaliana* ~75% < 65 bp; *P. patens* ~50% < 65 bp; p-value < 9.7E-165). Chung et al. (2006) provided initial evidence for a positive effect of the presence of 5’-UTR introns on gene expression. These findings and previous reports suggest that properties of 5’-UTR introns influence gene expression level, regulation, translation and nonsense-mediated mRNA decay [71,72]. As a consequence, the high percentage of genes with 5’-UTR introns, their unique length, and the fact that ~50% of *P. patens* loci with annotated alternative transcripts undergo alternative splicing in UTRs, suggests that the moss makes frequent use of this type of gene regulation.

### Improved functional annotation of moss proteins

The predicted V1.6 protein sequences were functionally annotated by homology transfer using BLAST2GO [73] and InterproScan [74]. Existing Gene Ontology Annotations (GOA) from previous releases were mapped, curated and integrated. GOA were extended using a novel in-house subcellular localization annotation pipeline called pred2GOA which allows the integration of GOAs from multiple sources by weighted combination of results from experimental evidence, subcellular target predictions and homology-based methods (see Methods for details) to improve GOA accuracy. The resulting non-redundant, transcript-wise *P. patens* V1.6 GOA contains 66,234 terms (Table 4). While we could increase the number of terms in the GO namespaces “biological process” (+4900 terms) and “cellular component” (+19,878 terms), the number is reduced for “molecular function” (-15,479 terms) in comparison to V1.2. However, comparison of both GOAs reveals a prominent set of protein kinase family proteins (248 sequences) with a very high number of assigned terms (in total 9176) in V1.2. Manual inspection identified these terms as false assignments. Excluding these false-positive terms leads to a general increase of terms and annotated gene products in V1.6 (Table 4). In total, 18,786 loci, i.e. 58% of all predicted protein-coding genes, have been assigned at least one GO term. This is a substantial improvement over V1.2 (41%). While we could increase the number of genes with a term for “biological process” (BP) by 2285 and “molecular function” (MF) by 2702, the number of genes with a “cellular component” (CC) term has increased by 10,065. A significant part of the assigned “cellular component” terms are based on supervised subcellular target predictions (pred2GOA, see Methods). To help scientists in evaluating the quality of annotations, we rely on the assignment of evidence codes [75], which offer a direct interpretation of the support for each GOA. After the automatic processing of terms, most of the annotations (~77%) is still supported only by IEA (Inferred by Electronic Annotation) followed by ISS (Inferred from Sequence or Structural Similarity) with ~23%. As noted earlier, only a few genes have annotation supported by experimental evidence. This now becomes obvious in the GOA - only 18 genes have evidence codes indicative of experimental support (0.04% of all assigned GO terms). With the development of our cosmoss.org community annotation service discussed below we hope to improve this situation and invite scientists to annotate their genes of interest. Building on and extending the GAF format, our databases and web service also offers additional information for every GOA describing the algorithmic or experimental source, references and crosslinks which can be used to further assess the quality of annotations.

**Table 4:** **Comparison of the Gene Ontology (GO) annotation of *P. patens *V1.2 and V1.6**

	**Total GO terms**	**BP terms**	**MF terms**	**CC terms**	**Genes with GO terms**	**Genes with BP**	**Genes with MF**	**Genes with CC**	**Protein-coding genes**
**V1.2**	56,935	10,681	39,894	6,360	11,586	8,449	10,408	4,774	27,966
					(41%)	(30%)	(37%)	(17%)	
**V1.6**	66,234	15,581	24,415	26,238	18,786	10,326	13,110	14,839	32,275
					(58%)	(32%)	(41%)	(46%)	

A general increase of protein-coding genes with an assigned GO term could be achieved in V1.6. The number of GO terms “molecular function” has been manually corrected for V1.2 (see text in section). BP – “biological process”: MF – “molecular function”; CC –“cellular component”.

The most common and useful application of GOA is the comprehensive functional analysis of experimentally defined gene sets by ontology term enrichment analysis [76]. The significance of results obtained by enrichment analysis strongly depends on the quality and depth of the underlying GOA. In order to assess the utility of the V1.6 GOA for exploratory enrichment analysis, we compared the Arabidopsis and Physcomitrella GOAs to test whether we could reproduce lineage-specific expansions previously discovered using phylogenetic and phylogenomics approaches ([10]; Table 5). In comparison to *A. thaliana,* gene families like flagellum associated dyneins [10,69], the light harvesting (LHC) superfamily including chlorophyll a/b binding proteins and early light-inducible proteins [10,13], histidine kinases and response regulators [10], phenylalanine ammonia lyases (PALs; [77]) and aldehyde dehydrogenases (ALDHs; [78]), are expanded in *P. patens.* Comparison of the underlying gene lists of the terms identified by GO enrichment analysis with those of the phylogenetic studies reveals that these specific expansions are also reflected by comparing functional annotations using statistical methods.

**Table 5:** **Selected GO categories: *P. patens *in comparison to *A. thaliana***

**Gene family**	***q-value***	***GO term id***	***GO term***	***P. patens***	***A. thaliana***
Two component system – histidine kinases and response regulators	6.91E-26	GO:0000160	two-component signal transduction system (phosphorelay)	124	78
	4.60E-24	GO:0018106	peptidyl-histidine phosphorylation	72	3
	1.03E-28	GO:0000155	two-component sensor activity	95	3
	1.34E-08	GO:0000156	two-component response regulator activity	86	35
LHCs	1.39E-09	GO:0009765	photosynthesis, light harvesting	45	21
flagellum	0.000155148	GO:0001539	ciliary or flagellar motility	10	-
	3.11E-05	GO:0019861	flagellum	19	-
	0.000496321	GO:0030286	dynein complex	12	-
PAL	0.000726755	GO:0006559	L-phenylalanine catabolic process	14	3
	0.010050079	GO:0016841	ammonia-lyase activity	17	5
ALDH	0.000574734	GO:0004365	glyceraldehyde-3-phosphate dehydrogenase (phosphorylating) activity	18	4

The GO enrichment analysis was performed using topGO with a q-value cut-off <0.05. Depicted are only GO terms overrepresented in *P. patens* and associated with the gene families reported to be expanded in *P. patens*.

In summary, we improved the GO annotation of *P. patens* gene products in general and demonstrate that the annotation can serve as a solid basis for comparative and exploratory analyses. Compared to *A. thaliana* (TAIR10), where only 7% of all genes have no GO annotation, there are still more than 40% of all loci without any GO annotation for *P. patens*, and those with annotation are mainly inferred by electronic annotation (IEA). While a significant fraction of this 40% probably comprises orphan genes, special focus should be placed on the improvement of the functional annotation of the *P. patens* protein-coding genes to unravel its full potential as a reference model organism. To facilitate such efforts we developed the cosmoss.org community annotation interface to browse and alter the *P. patens* annotation (see section genonaut) discussed below.

### Gene families

Gene families (clusters) were retrieved by clustering a multitude of sequences of Archaeplastida proteins (Additional file 5: Table A4) using OrthoMCL [79]. Parameters were optimized as described in Methods to target Archaeplastida gene families sensu strictu, i.e. families of genes that evolved by speciation and duplications after the divergence of the red/green lineage from a single gene in the last common ancestor. Multi-gene or superfamilies thus are split across multiple clusters. The number of *P. patens* loci in clusters is 32,733, while after the subtraction of the annotated number of 32,275 protein-coding loci, 458 fall into multiple clusters. Some of these cases are due to fragmentary or false structure predictions caused by fusion of two or more distinct gene loci into a single locus and will be resolved in future.

**Figure 5:**
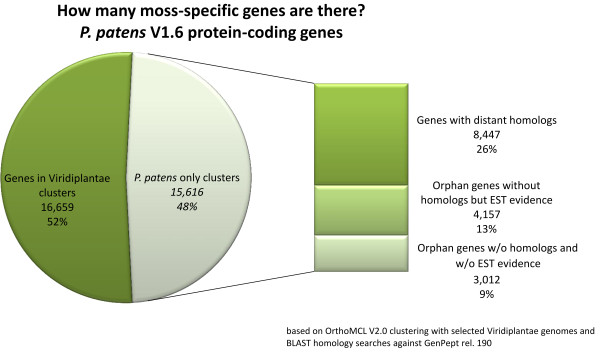
**How many moss-specific genes are there?** BLAST hits of *P. patens*-only clusters based on our OrthoMCL clustering with selected Viridiplantae genomes against GenPept (rel. 190). *P. patens* proteins were excluded from GenPept for this analysis.

**Figure 6:**
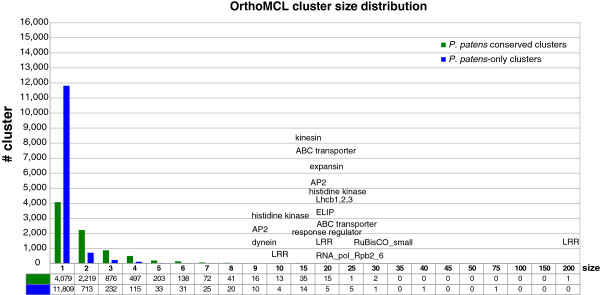
**Gene family sizes in *P. patens *proteins from several Viridiplantae were clustered using OrthoMCL.** Depicted are all protein clusters with regard to *P. patens* and sorted by cluster size. The clusters were subdivided into *P. patens* only clusters and clusters with at least one other member. Protein families found to be expanded in *P. patens* in comparison to *A. thaliana* are listed.

Strikingly, 48% of all *P. patens* loci are in Physcomitrella-only clusters (Figure 5), which independently supports an analysis based on protein domains where 52% of all *P. patens* genes have no Pfam domain [80]. Further filtering of these loci including BLASTP against GenPept and EST support indicates that ~22% (7169) of all loci in Physcomitrella-only clusters have no detectable homolog, while at least ~13% (4157) have no homolog but transcript evidence. These genes might represent true orphan genes, representing species- or lineage-specific adaptive innovations or non-protein-coding genes. The remainder falls into 8208 clusters with at least one other Viridiplantae protein (Figure 6). Thus, a prominent part of the *P. patens* gene complement could not be clustered with other plant proteins. In part, this could be explained by remaining fragmentary gene predictions and non-protein-coding genes. This is supported by the observation that the average transcript length of these *P. patens*-only singleton genes is less than 500 bp and the average number of exons is only two (total average is four exons, Table 3). However, at least 40% (4503) of these *P. patens*-only singleton genes have transcript evidence. Approximately two-thirds of all genes are in small clusters with less than three *P. patens* members. As already observed in the draft genome [10], several gene families are expanded by comparison to flowering plants. In total 7013 OrthoMCL clusters are shared by *P. patens* and *A. thaliana* containing 14,830 and 11,926 genes, respectively. Reported expansions in gene families [10], like LHCs (esp. chlorophyll a/b binding proteins), dyneins, histidine kinases and response regulators are also reflected by OrthoMCL cluster sizes (Figure 6). The majority of expanded families are in OrthoMCL clusters with more than five members in moss.

### Analysis of intron-loss and gain in the green lineage

Similar to 5’-UTRs, where we observe a striking number of multi-exon regions in moss, the overall number of single-exon transcripts is remarkable. While Chlamydomonas and Volvox contain fewer than 10% single-exon transcripts (Table 3 and Additional file 4: Table A3), this fraction is on average ~19% in other land plants, while moss possesses 23.4%. This may be due either to fragmentary gene predictions and residual non-protein coding genes, or may reflect secondaryintron losses.It has been observed that introns and their positions are highly conserved during land plant evolution [81]. In *A. thaliana* and *O. sativa* intron losses outnumber intron gains [82]. Furthermore, there is evidence for secondary intron loss; e.g. a moss sedoheptulose-1,7-bisphosphatase (*SBP*) gene which lost six out of seven introns [81]. Such intron losses might account for the relatively high percentage of single-exon genes in *P. patens*. One suggested mechanism for intron loss involves the reverse transcription of an mRNA followed by the (partial) replacement of the genomic DNA copy by an intron-less cDNA via homologous recombination, called retrocopying [83]. An increased rate of intron loss might thus be facilitated by the extraordinarily high rate of DNA repair by homologous recombination in *P. patens*. This hypothesis is based on models proposing a prominent role of gene conversion and DNA repair in intron loss [84,85].

Based on shared gene families the extent of secondary intron loss can estimated. There are 805 clusters with single-exon moss genes that have multi-exon homologs in other Viridiplantae. About half of them (404) contain also moss paralogs with exons in the same cluster. As some of these might be due to fragmentary gene models, we excluded models from the analysis that are significantly shorter than the median transcript lengths of non-Physcomitrella cluster members. A stringent way to define intron loss is to consider the median number of exons found in genes of other species in the same cluster. Based on these criteria, we find evidence for secondary loss of introns in about 14% (4405) of all *P. patens* genes (Table 6).

**Table 6:** Number of introns in Viridiplantae

	***C. reinhardtii***	***P. patens***	***O. sativa***	***A. thaliana***
Amount/fraction of intron-less genes	105 (0.7%)	941 (3%)	1,051 (3%)	1,196 (4%)
Amount/fraction of genes with less introns than median intron numbers of other plants	1,304 (8%)	4,405 (14%)	5,719 (14%)	5,158 (19%)

About 3% (941) of the *P. patens* genes seem to have lost their introns entirely, i.e. are of putative retrocopy origin, which is in the range of the other land plants (Table 6). The extension of the analysis to Arabidopsis, *Rice* and Chlamydomonas supports the findings from the comparison of absolute numbers of single-exon genes between algae and land plants described in the previous sections. The alga has significantly less (0.7%) single-exon genes than the three land plants (3-4%) under study. One likely scenario is an increased activity of transposons resulting in a secondary, maybe more recent, intron gain in algae [69,86]. This view is supported by the observation that intron positions are often not conserved between the two algae and the land plants (data not shown). Considering the comparable rate of intron loss in plant gene families, the fact that the total number of multi-exon transcripts in *P. patens* is similar to vascular plants, that only 60% of the 8979 single exon models are supported by expression evidence, and that more than half of these transcripts are shorter than 500 bp (which is less than half of the mean of *P. patens* transcripts, 1389 nt), leads us to conclude that a significant number of the predicted single exon genes represent fragmentary predictions, non-protein coding genes or pseudogenes.

### Gene family size evolution in Viridiplantae

**Figure 7:**
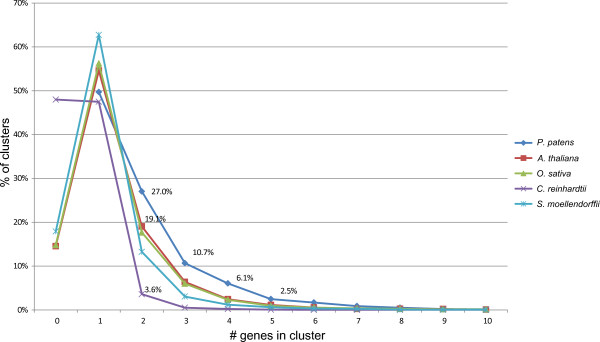
**Comparison of gene family sizes in conserved clusters distribution of genes per cluster and species common to *P. patens *and at least one other Viridiplantae (8208 cluster).**

We compared gene family size distributions along the green lineage by looking at all clusters containing *P. patens* and at least one other Viridiplantae species (8208 clusters/families; Figure 6). This analysis revealed that protein families with two to six members are more frequent in *P. patens* than in the other Viridiplantae (Figure 7), supporting the previously hypothesized balanced retention of paralogs that act as pseudoalleles in a haploid-dominant land plant [3]. This is most pronounced in clusters with two members, which amount to 27% in *P. patens* while being substantially less frequent in the other green organisms. In total, 832 clusters point to an expansion in *P. patens* in comparison to the other Viridiplantae. 184 of these *P. patens* gene families are more than twice the size of their largest green lineage counterpart.

Among these expansions house-keeping and metabolic gene functions are most prominent, independently supporting analyses of paralogs retained after the proposed whole genome duplication event ~45 million years ago [87] and of the unique presence of identical tandemly-arrayed genes [10]. Prominent examples of this expanded category of genes are abundantly expressed components of multimeric protein complexes like the ribosome and proteasome. In total, 86 of the clusters represent the different abundantly expressed protein components of the ribosome. On average these harbor ~1.5 times more genes in *P. patens* than in *A. thaliana*. For the 36 clusters representing structural components of the proteasome we observed on average 1.6 times the gene complement of Arabidopsis in the moss. These expansions are also detectable in the functional comparison of the two species using GO enrichment analysis (Additional file 6: Table A5).

In addition, families encoding for smaller complexes and monomers are expanded, including the Light Harvesting Complex II (LHCII) major antenna, Ribulose-1, 5-bisphosphate carboxylase oxygenase (RuBisCO) small subunit, TOC12 (translocase of the outer chloroplast membrane 12) and components of the splicing and translation machineries.

The expansion of house-keeping and metabolic gene functions is also mirrored by the findings of the GO enrichment analysis comparing *P. patens* and *A. thaliana* (Table 5 and Additional file 6: Table A5), which revealed an increased complement of genes involved in translation, oxidation-reduction, electron transport, microtubule based movement, glycolysis, and ATP synthesis. Additionally, specific expansions occurred which might represent lineage-/species-specific adaptations (e.g. expansins, MIKC*-type MADS box transcription factors, late embryogenesis abundant (LEA) proteins, early response to dehydration (ERD) proteins, cationic peroxidases) and in enriched GO “biological process” annotations (e.g. chitin catabolic processes, the phosphoenolpyruvate-dependent sugar phosphotransferase system, cell wall macromolecule catabolic processes, phosphatidylinositol-mediated signaling, chromatin assembly or disassembly, cell redox homeostasis, ciliary or flagellar motility and DNA repair). Some of these enriched categories like “ciliary or flagellar motility”, which can be explained by the absence of flagellated sperm in flowering plants, confirm the findings of previous analyses [10,69,88]. The majority of the above listed categories and families (nine out of 13) represents true novel insights which will help to unravel the much-cited qualities of the mosses in coping with abiotic and biotic stressors like their unique ability to repair DNA damage [89-91].

### Extension of the cosmoss.org resource to provide a permanent, central model organism database and annotation repository for *P. patens*

Protein-coding gene models were clustered to loci and the resulting locus definitions were used to derive information-rich locus identifiers (Cosmoss Gene ID; CGI; see Additional file 7: S2 and Additional file 8: Figure A2, and [92] for details).

With the development of genonaut [93], we have extended cosmoss.org by the capability to annotate *Physcomitrella patens* genes with regard to gene name, product name, description, and Gene Ontology (GO) terms. The interface which allows searching, browsing and editing of annotation is modular and can be extended to support additional (ontology) annotations as gene features. Traceability of annotations in terms of author and experimental evidence is crucial for quality assessment of information retrieval. Thus, the genonaut interface accepts the alteration of an existing gene description only if the source is specified. Integration of multiple sources into a unique abstraction layer is achieved by assigning unique and permanent Cosmoss Reference IDs (CRID) to author statements from all sources. The highest quality author statements are experimental evidence provided as references to peer-reviewed publications. The easiest way to achieve this is to provide a valid PubMed ID and the system automatically retrieves all relevant information from NCBI PubMed. If the source is a publication that is not tracked in PubMed, a custom reference can be created. If no publication is available, as the “weakest” possible evidence, a note in form of a text comment or web link describing the evidence is required. Besides the references the genonaut interface allows to link to other resources via database cross-references (Dbxref).

The gene products can be further annotated using GO terms. To allow convenient manual GOA, the genonaut interface assists the annotator by allowing the user to browse the appropriate GO namespace by keywords to assign the correct terms. In addition to the mandatory reference for a genonaut annotation, we have integrated the assignment of the GO evidence codes [75]. In this way the quality of each assigned GO term is directly discernible. As traceability is crucial for the maintenance of annotation quality, the genonaut system traces every annotation change using a history system. Thus it is possible to trace changes and possibly revert to a previous state if needed, but more importantly to comprehend the annotation history of every gene and annotation version.

Whereas the annotation browser capabilities are publicly available, the editor functions are restricted to registered cosmoss.org users (December 2012: 228 annotator accounts). The cosmoss.org curator team acts as a superior authority which supervises and validates the user provided annotations by direct personal communication.

Moreover, the genonaut interface provides a starting point to retrieve detailed annotation about *P. patens* protein-coding genes. Besides the possibility to search and edit the annotations and annotation history, the genonaut interface is linked to the sequence retrieval, genome browser and sequence viewer providing transcript and protein domain annotations.

To support the manual curation of gene structures we have integrated and adapted the Apollo structural gene annotation editor [94] for the cosmoss.org genome browser. Generated user_models (December 2012: 830 manually curated transcripts) are assigned CGIs (Additional files 7 and 8) from the user model namespace extended using the authors username to allow multiple versions per locus (e.g. Pp1s275_35**U2** ➔ Pp1s275_35**U2__zimmer**.1).

## Conclusion

Here we describe the complete re-annotation of the *P. patens* V1 genome assembly comprising structural and functional annotation of protein-coding genes and, for the first time, description of non-protein loci including tRNA, rRNA, miRNA, snoRNA and snRNA loci. Compared to V1.2 the improved structural annotation V1.6 resulted in 8387 additional protein-coding loci, 11,242 more complete genes and only 1582 unaltered gene structures. 70% of the 32,275 protein-coding genes are supported by EST evidence. Nearly half (~49%) of the protein-coding loci in V1.6 are now be considered complete, containing both UTRs. Furthermore, the information-rich cosmoss.org locus IDs also carry information on the chromosomal/scaffold localization and about alternative splicing of transcripts.

We significantly increased the number of genes with functional annotations (58% as compared to 41% in V1.2) in form of GO term annotations (GOA). Our quality assessment of the V1.6 GOA demonstrates sufficient annotation depth to recover results from previous high-quality phylogeny-based approaches using ontology term enrichment analysis. Nevertheless, there are still 41% of all loci without any GO annotation and only 0.04% of all assigned GO terms are supported by direct experimental evidence. Although this is a common phenomenon for most available plant genomes, special focus needs to be placed on the improvement of the functional annotation of the *P. patens* protein-coding genes in order for it to serve as a reference model organism and plant “flagship”. With the development of the cosmoss.org community annotation services described here allowing users to browse, view and alter functional and structural annotations of moss genes, transfer and exchange of knowledge is greatly facilitated. Including the described extensions of the resource, cosmoss.org now is well-equipped to serve as a permanent, central model organism database and annotation repository for *P. patens*.

We demonstrate the utility of the provided annotation and resources for the comparative study of plant evolution including the analysis of codon usage (see Additional file 7: S1 and Additional file 9: Figure A3), alternative splicing, gene structure evolution as well as the detection of lineage- and species-specific expansions of gene families and biological processes.

Results from our comparative analyses were mostly consistent with previous observations, but also provided several novel insights. In particular, we found further evidence for intron loss during land plant evolution and secondary intron gain in the alga Chlamydomonas. Investigation of alternative splicing and gene structures revealed a unique complexity of 5’-UTRs in the moss, pointing to the importance of UTRs for the regulation of gene expression in this early diverging land plant.

Our comparative analysis of functional annotations and protein clusters revealed expansions of moss house-keeping and metabolic gene functions as well as hitherto unknown lineage-specific expansions. In total, 832 gene clusters are expanded in *P. patens* and at least ~13% of all gene loci are orphan genes as they have no homolog in other as yet published genomes. Subsequent functional analysis of this data set will further extend our understanding of the unique capabilities to cope with abiotic and biotic stressors and to efficiently repair DNA damage.

## Methods

### *P. patens* reference genes

In total, 137 manually annotated and validated *Physcomitrella patens* gene structures are in the cosmoss.org genome browser track “Ppref genes”. Some of the genes are directly derived from published genes (GenBank) or provided by the scientific community, but the majority was extended or corrected manually using ESTs and FLcDNAs. In addition, in-house validated sequenced *P. patens* gene structures were added.

### EuGène *P. patens* gene prediction process

EuGène [58] allows combining various type of evidence including e.g. ESTs, mate pair information, homologous sequences, existing gene predictions and splice site predictions. Our EuGène predictions for *P. patens* are based on a splice site prediction using SpliceMachine [57] trained on filtered *P. patens* EST alignments, coding, intronic and intergenic regions, homology evidence (*A. thaliana* homologs) and are filtered using transposon related sequences and gaps. The optimal parameters were determined on an independent *P. patens* reference genes set not used for training of EuGène. Two *P. patens* whole genome EuGène predictions went into generation of the release V1.6. The first contains 37,872 predicted loci and was restricted to generate UTR regions only if transcript evidence is available and the second contains UTR regions predicted *ab initio* (46,071 loci).

The first EuGène model predicts ~94% of all CDS exons in the reference genes correctly, whereas the second does so for ~95% (results are summarized in Table 3). Both predict 76% of all reference CDS without any error. That implies that the EuGène predictions perform well in predicting the exons but split several loci into two or more distinct genes. While manually inspecting these problematic loci we have noticed that gene models created by the JGI [10], which were not been selected for release V1.1, were often better than the selected model model, or could be used to overcome, or respectively complement, the EuGène predictions. As the method of choice to combine all available *P. patens* evidence and to further improve the protein-coding gene structures we have used EVidenceModeler [59].

### EvidenceModeler (EVM) - weighted consensus gene model predictions

EVM (Haas et al. 2008) combines evidence from different sources into a consensus gene structure prediction. With the possibility to weight and unite the different evidence and optimize their combination, EVM utilizes the different sources by equating the drawbacks of individual sources but also boosting their strong points. For *P. patens* we have used EVM to find the optimal combination of PASA [60] transcript assemblies, EST alignments and five different whole genome protein-coding gene predictions. The process is also described in Additional file 3: Figure A1. The resulting models were subsequently subjected to PASA to model the UTR regions. As a consequence, all UTRs in release V1.6 are supported by transcript evidence. The utilization of EVM has enabled us to increase the prediction performance on the reference gene set (86.1% of all CDS and 97.3% of all CDS exons are correct; see Additional file 2: Table A2).

### Additional gene structure predictions using EuGène

EuGène [58] version 3.4 was adapted and trained for *P. patens* on the basis of the Ppref genes set (mentioned above) and including a species-specific splice-site prediction and IMM (Interpolated Markov Model) models trained on intergenic, CDS and UTR regions. Gaps in the genomic sequence and repetitive regions, in particularly LTR retrotransposons [10], were masked for the training and predictions. As additional evidence, homologous protein sequences (Swissprot rel. 13.4 and *Arabidopsis thaliana* TAIR7 [34] homologs) and in particular EST alignments 360,974 from GenomeThreader [61], 118,243 from sim4 [95] and 97,373 from exonerate [96] were used. If available, we also provided EST mate pair information into EuGène (63,945 EST mate pairs). Two whole genome EuGène predictions were used for the consensus model approach leading to V1.6. The training input of these two models was the same except that one model was additionally trained with 5’- and 3’-UTR regions (EuGène MarkovIMM plugin).

### Splice site prediction

*P. patens* EST alignments were loaded into a Bio::DB::Seqfeature database. The exclusion of alternative splicing and bad quality EST alignments led to a distinct, species-specific splice-site training set for SpliceMachine. Splice sites were only taken into account where GenomeThreader [61], sim4 [95] and exonerate [96] did exactly the same EST alignment. Two models, one donor (GT) and one acceptor (AG) model have been generated and have been used for EuGène training.

### PASA – transcript assemblies

PASA [61] assemblies were performed as directed on the software homepage [97], with the following modifications: Per default PASA uses GMAP [62] for transcript alignments, however, our evaluation process (data not shown) reveals improved EST alignments using GenomeThreader. PASA offers the possibility to include alignments in GFF3 format via the --IMPORT_CUSTOM_ALIGNMENTS_GFF3 switch. The *P. patens* GenomeThreader EST alignments were converted into PASA compliant GFF3 format. PASA per default supports only one alignment per EST/transcript. Therefore with regard to duplicated genes (especially tandemly arrayed genes and (near-)identical genes) the corresponding EST alignments were renamed. E.g. the sequence ppsp14d22fl matches to 2 loci in the genome, so the corresponding sequence is duplicated and renamed: ppsp14d22fl and ppsp14d22fl_2. Transcript alignments with less than 90% EST length coverage or less than 95% alignment identity were discarded. The maximum allowed intron length was set to 20,000 nt, based on the longest observed intron supported by Sanger ESTs. Three cycles of PASA annotation loading, annotation comparison, and annotation updates were used to maximize the incorporation of transcript alignments into the transcript assemblies.

### Consensus gene predictions – unfiltered V1.6 models

The *P. patens* JGI AllModels V1.1 (http://ftp.jgi-psf.org/pub/JGI_data/Physcomitrella_patens/v1.1/transcripts.Phypa1_1.AllModels.fasta.gz) and the cosmoss.org EuGène models, described in the previous section, and transcript alignments (PASA, exonerate and sim4) were combined and weighted with EVidenceModeler (EVM). EVM combines all evidence per locus into one consensus gene structure model. EVM was trained on the Ppref genes deducting reference genes used for the training of EuGène. Subsequently the UTR regions were modeled by *P. patens* PASA transcript alignment assemblies as described on the PASA homepage [97]. In this context models with alternative splicing evidence were generated and incorporated into V1.6.

### Functional gene annotation

The unfiltered V1.6 gene models and the corresponding predicted protein sequences, respectively, were subjected to BLAST2GO [73]. The initial BLASTP (e-value cut-off: 1E-4) search was performed against a *P. patens* V1.1 subtracted GenPept release 172.0 to allow an unbiased functional annotation, independent from the previous entirely automatic V1.1 GOA. For the BLAST2GO annotation step the minimum coverage between a hit and its HSP was set to 40%. The validation step as well as the integration of InterProScan V4.5 (InterPro release v22.0) based GO annotation was used to generate the GOA for the *P. patens* V1.6 proteins.

GO term annotation was extended by experimental evidence, various subcellular target predictions and homology-based methods using the pred2GOA method described below. Existing functional annotations like gene names, description lines, GO terms and KEGG EC numbers and KO terms were collected from JGI and Kyoto Encyclopedia of Genes and Genomes [98] and combined into a non-redundant database. Existing experimental evidence for subcellular localization was also manually integrated as GOA if available. Additional functional annotations were created using the homology-based methods BLAST2GO, IPRScan [74] and KAAS [99]. These two steps resulted in GO terms with IEA evidence codes. Subcellular localization of the protein sequences was predicted considering the individual gene’s full-length status using several tools: TAPScan [100], MultiLoc [101], WolfPSORT [102], TargetP [103], ChloroP [104], SignalP [105], Prosite KDEL [106], HMMTOP [107] and MEMSAT3 [108]. To combine these predictions with the existing GOA, we developed the algorithm pred2GOA which allows weighted integration of GOA from multiple sources. Resulting predictions were translated into cellular component GO terms and compared to the existing GOA at the GO slim (plants) level. The assignment of GO terms is based on a weighted majority rule consensus. If the underlying gene did not have an annotated UTR and a start codon, at least one of the predictors’ predictions had to be based on more than the N-terminal region of the protein. Resulting GO term assignments were reviewed for consistency and defined as “Inferred from Sequence or Structural Similarity” (ISS). The resulting GO annotation was mapped to GO slim terms using the Blast2GO internal mapping function using the “goslim_plant.obo” ontology subset.

### GO enrichment analysis

The enrichment analyses were performed using the Bioconductor package topGO [109]. We used and compared results from both the classical Fisher’s Exact Test and the topGO algorithm “weight01” for test statistics with a p-value cut-off 0.05. The *Arabidopsis* GOA was downloaded from the TAIR ftp server (TAIR10; http://ftp.arabidopsis.org/Ontologies/Gene_Ontology/ATH_GO_GOSLIM.txt).

### Protein clusters and representative gene model selection

Although certainly useful in single gene analysis and the study of AS, the use of all splice-variants in large-scale comparative analysis introduces undesirable complications. Therefore gene catalogues are usually reduced to one representative isoform per locus prior to large-scale analyses. By convention, the representative model is the variant with the lowest splice variant index. A representative gene model per locus was selected by the following procedure: At the first step, all *P. patens* V1.6 proteins together with the proteins of various sequenced Archeaplastida (Additional file 5: Table A4) were clustered using OrthoMCL V.2 [79]. The inflation parameter was optimized using a set of reference gene families (e.g. [10,15]). The inflation value was set to 1.3. The resultant clusters were aligned with MAFFT ginsi v6.712b [110] and subsequently the uncorrected pairwise distances were calculated with distmat (EMBOSS 6.1.0; [111]). The distmat matrices were used to select the *Physcomitrella* model as representative for a locus that has the least of all substitutions (minimum distmat distance) to any non-*Physcomitrella* cluster member. Clusters with only *Physcomitrella* sequences were subjected to a BLASTP search against GenPept (release 172.0). In this case the representative per locus was set to the *Physcomitrella* sequence which covers its closest GenPept hit, in terms of alignment length, best. If still no clear representative could be determined, the model with the longest transcript length was chosen as the representative. Due to the fact that the clustering is based on sequence information alone, not all splice variants per locus were grouped into the same cluster in some cases. Thus, the number of loci in clusters slightly exceeds the number of physical loci.

### RNA genes

Pre-tRNA genes were predicted by combining results from tRNAscan-SE 1.21 [112] and ARAGORN 1.1 [113]. rRNA loci were predicted using RNAmmer-1.2 [114] and extended by mapping the available SILVA *Physcomitrella* rRNAs [115] to the genome with BLASTN.

Non-protein coding loci and gene families where determined using Infernal [116] with the RFAM (version 8.1) [117] covariance models and by integration of the miRBase 18 [51]*Physcomitrella* miRNA classifications and annotations.

### *P. patens* V1.6 protein-coding genes filtering

On the basis of a Bio::DB::SeqFeature database [118] the V1.6 gene models were filtered against the annotated LTR-retrotransposons [10], Repbase (RELEASE 20080801; [119]) using RepeatMasker v3.26 [120] and the non-coding RNA described in the previous section.

### Codon usage

The effective number of codons was calculated with CodonW 1.4.4 [121].

### Statistics

Fisher’s exact tests and Wilcoxon tests were performed with R 3.0.0. The p-values were corrected for multiple testing using fdr.

### Availability of supporting data

The complete annotation and sequence information comprising: Gene structure releases in GFF3 format: gene, transcript, CDS, protein, UTR sequences in FASTA format; Mappings/lookup tables, GO annotations in GAF format are accessible via the cosmoss.org download section [122].

## Competing interests

The authors declare that they have no competing interests.

## Authors’ contributions

ADZ, SR carried out the protein-coding gene structure predictions and annotation. YP supervised the EuGène training and prediction. DL performed non-protein coding gene annotation. ADZ and DL developed the functional annotation pipeline, performed the functional annotation, and drafted the manuscript. DL and ADZ conceived, supervised and designed the curation process, annotation interfaces and database backends. KB implemented the genonaut web interface. TN and MH generated and contributed FLcDNA sequences, discussed results and helped in writing the manuscript. RR and SAR supervised the study and contributed to writing the manuscript. All authors read and approved the final manuscript.

## Supplementary Material

Additional file 1: Table A1 *P. patens* transcript evidence. Click here for file

Additional file 2: Table A2 Gene prediction performance on *P. patens* reference genes. Click here for file

Additional file 3: Figure A1 Annotation process overview chart. Click here for file

Additional file 4: Table A3 Protein-coding gene statistics of selected Viridiplantae. Click here for file

Additional file 5: Table A4 Archeaplastida genomes sources. Click here for file

Additional file 6: Table A5 topGO Gene ontology (GO) enrichment analysis between* P. patens* and *A. thaliana*. Click here for file

Additional file 7 Supplementary_text - Codon usage & Protein-coding locus definition. Click here for file

Additional file 8: Figure A2 Gene ID naming – Cosmoss.org gene identifiers. Click here for file

Additional file 9: Figure A3 Codon usage of *P. patens* and *A. thaliana* transcripts. Click here for file
