# Supplementary figures and images for: Reannotation and extended community resources for the genome of the non-seed plant Physcomitrella patens provide insights into the evolution of plant gene structures and functions
Source: BMC Genomics. 2013 Jul 23;14:498. doi: 10.1186/1471-2164-14-498 (PMC3729371; doi:10.1186/1471-2164-14-498)

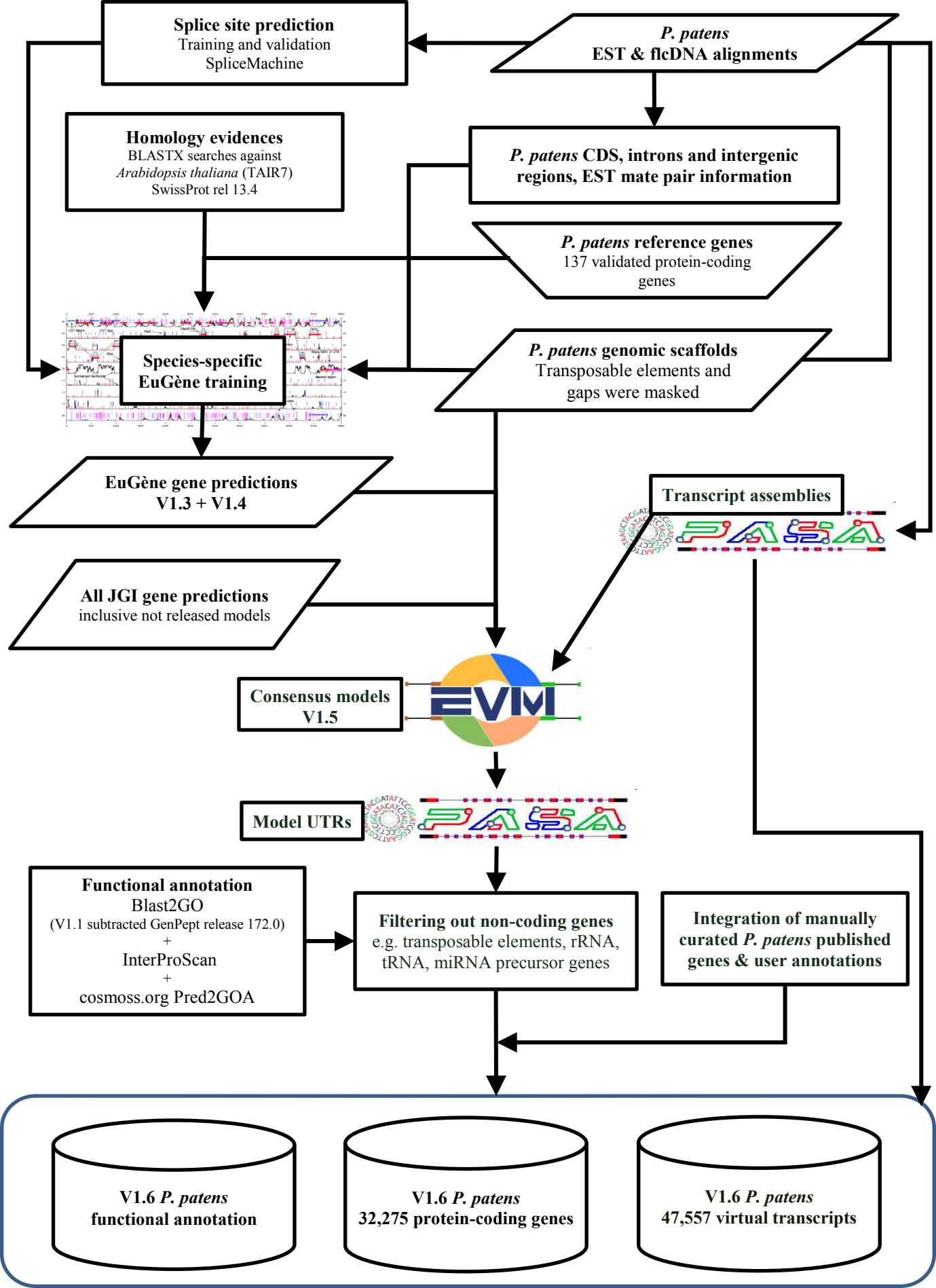

Supplement: Additional file 3: Figure A1 — Annotation process overview chart. [file 1471-2164-14-498-S3.pdf]

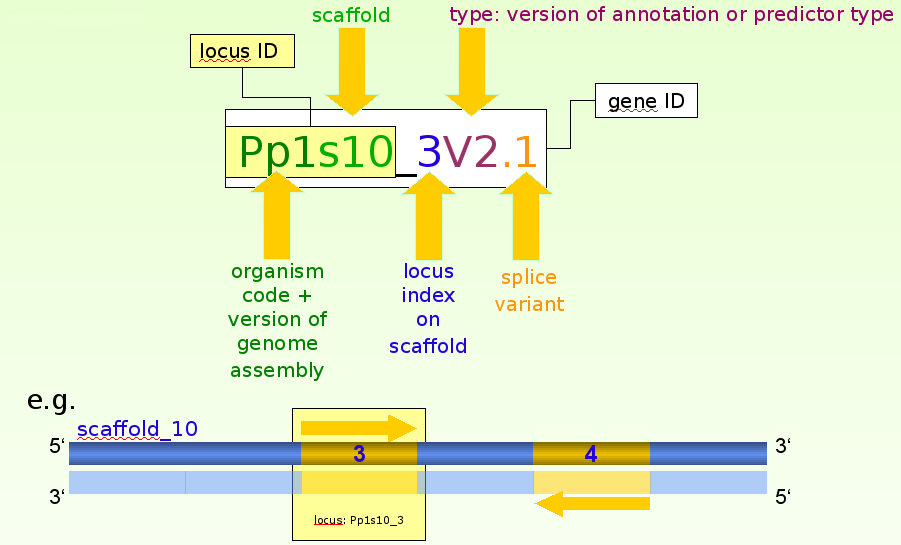

Supplement: Additional file 8: Figure A2 — Gene ID naming – Cosmoss.org gene identifiers. [file 1471-2164-14-498-S8.tiff]

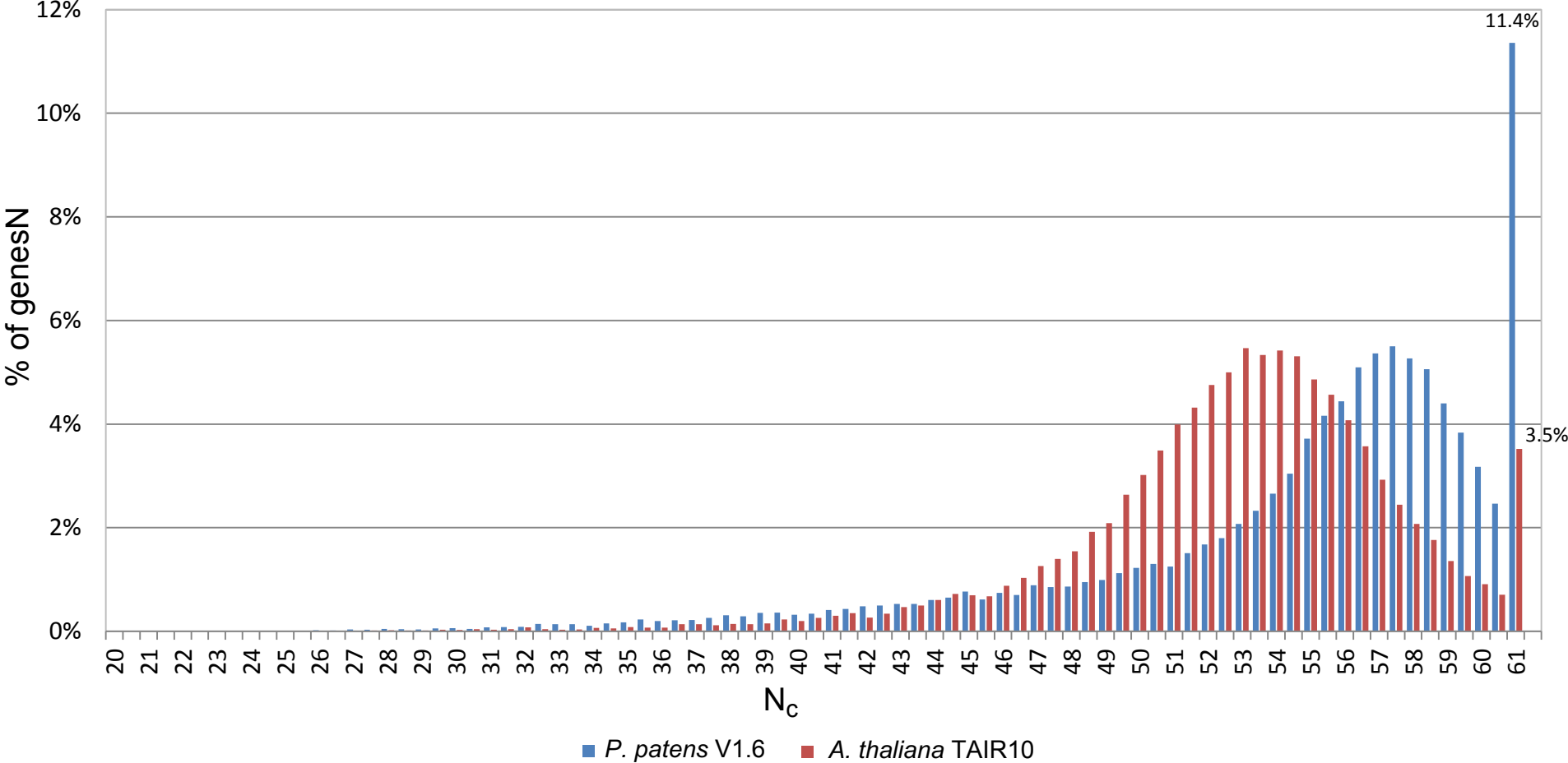

Supplement: Additional file 9: Figure A3 — Codon usage of P. patens and A. thaliana transcripts. [file 1471-2164-14-498-S9.pdf]
