# Supplementary material for: Reannotation and extended community resources for the genome of the non-seed plant Physcomitrella patens provide insights into the evolution of plant gene structures and functions
Source: BMC Genomics. 2013 Jul 23;14:498. doi: 10.1186/1471-2164-14-498 (PMC3729371; doi:10.1186/1471-2164-14-498)
Supplement: Additional file 7 — Supplementary_text - Codon usage & Protein-coding locus definition. [file 1471-2164-14-498-S7.docx]

## S1 Codon usage

Previous analyses based on virtual transcripts from assembled EST evidence [10] estimated 15% of the transcripts showed no codon bias. In V1.6 we still find a high percentage of transcripts (11.4%) that have no codon bias at all. The evaluation on the full genomic scale confirms the previously [10] described data in that we observe a similar distribution as for *Arabidopsis* (TAIR10; [73]), but shifted towards genes with no codon bias (figure A2). Over 50% of all *P. patens* transcripts have an effective number of codons (N_c_) of 56.5 or higher. The observed codon usage preferences reinforces the development of the moss as an excellent plant system for heterologous gene expression, e.g. of human genes [74, 75].

## S2 Protein-coding locus definition and information-rich gene identifiers

The regions of protein-coding loci were defined based on all overlapping gene predictions (JGI V1.1 and cosmoss.org predictions) per locus. To achieve this, all overlapping gene predictions on a scaffold were clustered. In a second step, the obtained pre-loci were sub-clustered using a single linkage approach. The clustering was based on exon overlaps requiring coverage of at least 50% of the longer sequence or at least 20% of the longer and at least 80% of the shorter sequence. The derived locus definitions provide technically and biologically important information on the overlapping gene predictions. To access this information, the resulting loci were assigned a unique Cosmoss Gene ID (CGI). A summary of the locus naming conventions is illustrated in Figure A3 and is documented in the *P. patens* annotation guidelines [107].

These identifiers have an information-rich syntax carrying all important data about a gene model. The first part consists of **Pp** for *P. patens* and the assembly version (**Pp**1 → V1 assembly). The assembly version is followed by the **s**caffold number preceded by an **s**, e.g. **Pp**1**s10**. Once the *P. patens* scaffolds are replaced by the scaffolded pseudochromosomes, this field can be substituted with the chromosome number e.g. Pp**2c10** (assembly version 2, chromosome 10). Following an underscore are the locus IDs that are numbered consecutively per scaffold/chromosome. E.g. Pp1s10**_3** reads as *P. patens* gene model 3 on scaffold_10 in the assembly version 1. Every predicted gene model is labeled according to its prediction source or the respective annotation version the model was released with. All cosmoss.org and JGI gene predictions were encoded as prediction sources based on the first character of the tool used for prediction and an index to avoid ambiguity (EVidenceModeler = E4). Gene models that are part of a released gene catalogue (e.g. V1.2 = V2) are also accessible via a CGI specific for the respective release. E.g. the gene product Pp1s10_3**V2**.1 derives from the gene structure Pp1s10_3V2 from locus Pp1s10_3. The release-specific CGI is used as the primary accession number to retrieve and unambiguously identify loci, full gene structures, proteins and transcripts. AS is incorporated into the CGI convention by appending splice variant indices - e.g. Pp1s10_3E4**.2** stands for a gene prediction on scaffold 10, locus 3, is generated by EVidenceModeler and represents the second (**.2**) splice variant at this locus. As described in methods, splice variants are ordered to indicate the most representative variant as **.1**. While the inherent locus definitions convey the biological concepts of physical location on the DNA as well as splice variants, the CGI nomenclature also provides technical information which allows discerning assembly and gene prediction versions directly from the identifier. Furthermore, for released models, mapping between different gene annotation releases per assembly is possible by changing the number of the version field (e.g. Pp1s10_3**V2** → Pp1s10_3**V6)**. Locus IDs are assigned incrementally per scaffold . Future releases at chromosome scale will adopt an incrementation scheme of similar to TAIR (+100), i.e. allowing the introduction of yet undiscovered, intervening gene loci e.g. by manual annotations.
